# Cancer among circumpolar populations: an emerging public health concern

**DOI:** 10.3402/ijch.v75.29787

**Published:** 2016-01-12

**Authors:** T. Kue Young, Janet J. Kelly, Jeppe Friborg, Leena Soininen, Kai O. Wong

**Affiliations:** 1 School of Public Health, University of Alberta, Edmonton, Alberta, Canada; 2 Alaska Native Epidemiology Center, Alaska Native Tribal Health Consortium, Anchorage, AK, USA; 3 Department of Clinical Oncology, Rigshospitalet, Copenhagen, Denmark; 4 Department of Public Health, University of Helsinki, Helsinki, Finland

**Keywords:** cancer, Arctic, epidemiology, prevention, Indigenous people, Inuit, North American Indians, Sami

## Abstract

**Objectives:**

To determine and compare the incidence of cancer among the 8 Arctic States and their northern regions, with special focus on 3 cross-national indigenous groups – Inuit, Athabaskan Indians and Sami.

**Methods:**

Data were extracted from national and regional statistical agencies and cancer registries, with direct age-standardization of rates to the world standard population. For comparison, the “world average” rates as reported in the GLOBOCAN database were used.

**Findings:**

Age-standardized incidence rates by cancer sites were computed for the 8 Arctic States and 20 of their northern regions, averaged over the decade 2000–2009. Cancer of the lung and colon/rectum in both sexes are the commonest in most populations. We combined the Inuit from Alaska, Northwest Territories, Nunavut and Greenland into a “Circumpolar Inuit” group and tracked cancer trends over four 5-year periods from 1989 to 2008. There has been marked increase in lung, colorectal and female breast cancers, while cervical cancer has declined. Compared to the GLOBOCAN world average, Inuit are at extreme high risk for lung and colorectal cancer, and also certain rare cancers such as nasopharyngeal cancer. Athabaskans (from Alaska and Northwest Territories) share some similarities with the Inuit but they are at higher risk for prostate and breast cancer relative to the world average. Among the Sami, published data from 3 cohorts in Norway, Sweden and Finland show generally lower risk of cancer than non-Sami.

**Conclusions:**

Cancer among certain indigenous people in the Arctic is an increasing public health concern, especially lung and colorectal cancer.

The 8 member states of the Arctic Council (Canada, Denmark, Finland, Iceland, Norway, Sweden, Russian Federation and the United States, hereafter referred to as the Arctic States) comprise some of the world's economically most developed countries. Yet, within these countries, substantial health disparities persist, between northern and southern regions, and within the North, between indigenous and non-indigenous peoples, although the extent of the disparities varies across countries (1–3).

We report on an epidemiological review of cancer in the Arctic States and their northern regions. Cancers such as lung and breast can be viewed as an indicator of the rapid social, economic and environmental changes that Arctic populations, especially indigenous peoples, are experiencing. A circumpolar comparative framework is particularly useful as countries and regions that share many commonalities can learn best practices from one another in cancer prevention and control.

Previous reviews have focused on specific regions such as Greenland (4), Alaska (5), northern Canada (6), and specific populations such as the Inuit (7,8). The current review compares all northern regions within the Arctic (for which data are available), with special focus on 3 indigenous populations whose traditional homelands span across present day national boundaries – Inuit, Athabaskans and Sami. We also offer a global perspective by putting the circumpolar populations in the context of major geopolitical regions of the world.

## Methods and data sources

We obtained data on cancer incidence among the Arctic States and their northern regions from national statistical agencies, cancer registries and regional health authorities. Such data refer to the total populations with all ethnicities combined. These agencies are listed in the notes of Table I. There are also international cancer databases such as NORDCAN for the Nordic countries (9) and GLOBOCAN for the member states of the World Health Organization (10). With the exception of Russia, the Arctic States have national cancer registries that are considered to be of sufficient quality to be included in the International Agency for Research on Cancer's statistical compendium *Cancer Incidence in Five Continents*, CI5 (11). The St. Petersburg registry is the only Russian entry in CI5. National and regional cancer incidence data from Russia are available from the annual reports of the P.A. Hertzen Research Institute of Oncology in Moscow (12). In recent years, Norwegian international assistance efforts have been directed at improving the quality of the cancer registry of an Arctic region in north-western Russia (13). In this study we did not access data from this registry.

**Table I:** Mean age-standardized incidence rates of selected cancer sites in 8 Arctic States and their northern regions, 2000–2009

		All sites		Lung		Colon/rectum		Breast	Cervix	Prostate
					
Arctic state/Northern region	Mean population	M	F	M	F	M	F	F	F	M
United States	294,366,300	369.9	291.6	54.3	36.6	36.4	26.4	89.9	6.7	107.1
Alaska	662,061	361.9	303.1	52.9	39.0	37.7	29.1	96.9	6.7	104.4
Canada	32,143,213	332.7	273.7	48.9	34.3	42.4	28.6	80.0	6.3	88.9
Yukon	31,697	269.4	248.9	45.2	30.8	41.3	33.6	76.0	5.7	58.0
Northwest Territories	42,633	280.8	271.2	45.2	36.8	63.6	52.2	88.0	6.1	64.5
Nunavut	29,982	327.4	381.5	145.8	155.3	58.0	72.1	42.7	10.5	19.5
Denmark	5,417,146	320.4	306.5	45.4	34.5	42.4	32.0	92.5	10.4	60.4
Greenland	56,577	317.2	304.2	98.7	63.1	36.5	35.4	44.4	23.7	16.9
Faroe Islands	47,781	228.0	221.9	23.6	14.7	31.9	29.4	65.1	11.7	44.3
Iceland	298,582	310.1	282.2	34.0	32.0	32.0	23.9	87.9	8.5	95.6
Norway	4,628,970	323.8	271.5	36.3	22.7	42.7	34.7	75.1	9.5	92.9
Nordland	236,639	324.4	284.7	36.8	25.0	44.2	34.7	72.0	13.7	60.2
Troms	153,281	317.1	262.7	39.4	22.0	40.2	30.6	69.3	11.8	57.0
Finnmark	73,157	287.9	250.8	48.4	26.8	63.7	26.0	64.2	10.1	63.1
Sweden	9,042,112	279.8	244.0	20.3	16.8	31.2	24.3	80.2	7.0	101.3
Västerbotten	256,679	269.9	243.5	16.0	12.7	33.0	26.4	81.4	5.3	99.4
Norrbotten	252,754	220.5	219.2	14.8	14.5	23.3	19.6	70.5	7.7	73.4
Finland	5,245,935	287.7	234.5	34.6	10.8	26.9	19.9	83.2	4.1	94.7
Pohjois-Suomi	464,704	253.9	208.8	36.6	10.1	20.7	16.8	68.1	3.3	77.5
Lappi	187,033	271.0	208.3	38.6	12.2	21.8	15.3	68.4	4.3	89.8
Russia	143,784,868	267.6	192.8	58.6	6.9	27.6	20.2	40.7	12.0	20.1
Murmansk Oblast	877,503	300.9	212.2	67.4	7.7	38.6	26.4	45.7	11.1	26.9
Kareliya Republic	705,600	276.1	183.6	68.3	4.7	34.1	22.4	39.9	15.5	21.5
Arkhangelsk Oblast	1,310,255	285.3	188.8	67.0	6.0	30.1	23.5	35.0	10.7	20.5
Komi Republic	998,158	293.2	192.4	71.6	9.2	35.7	23.9	36.8	14.4	15.6
Sakha Republic	951,425	256.5	183.2	59.5	19.8	21.4	18.8	30.2	13.5	8.0
Magadan Oblast	176,652	321.3	209.5	74.5	14.2	35.3	27.9	43.3	16.3	15.3
Chukotka AO	52,399	268.7	224.1	60.9	23.4	26.0	35.8	40.7	19.7	10.6

Notes:

All rates are directly standardized to the IARC World Standard Population and expressed as per 100,000. Rates refer to the total national/regional population with all ethnicities combined.

Data for Denmark do not include Greenland or the Faroe Islands. For all other Arctic States, data for the northern regions are part of the national data.

Northern regions refer to:

United States – the State of Alaska.

Canada – the 3 territories north of the 60^o^N latitude – Yukon, Northwest Territories and Nunavut.

Kingdom of Denmark – the self-governing territories of Faroe Islands and Greenland.

Norway – the county (*fylke*) of Nordland, Troms and Finnmark.

Sweden – the county (*län*) of Västerbotten and Norrbotten.

Finland – the regional state administrative agency (*aluehallintovirasto* or AVI, formerly *lääni*) of Pohjois-Suomi and Lappi

Russia – various republics, oblasts and autonomous okrugs; Due to administrative changes, data on several Russian Arctic autonomous okrugs [AO] with a significant indigenous population are not available: Nenets AO, Yamalo-Nenets AO, Khanty-Mansi AO, Taymyr AO, Evenki AO, Koryak AO.

Data sources:

United States – based on the National Program of Cancer Registries and retrieved from the CDC Wonder website of the Centers for Disease Control and Prevention.

Canada – based on the Canadian Cancer Registry of Statistics Canada (CANSIM and custom tabulations by special request.

National data for Denmark, Iceland, Norway, Sweden and Finland are from NORDCAN on the web v5.0.

Regional data for Norway, Sweden and Finland for the 2000–2004 period are from PC-NORDCAN v2.4, and for the 2005–2009 period are from the Norwegian, Swedish and Finnish Cancer Registry respectively.

Greenland and Faroe Islands – based on background tables for the annual publication *Health Statistics in the Nordic Countries* published by the Nordic Medico-Statistical Committee NOMESCO.

Russia – based on tables published in the annual report *Malignant Neoplasms in Russia (Incidence and Mortality)* published by the P.A. Hertzen Research Institute of Oncology in Moscow. Only 2007 and later years are available online; earlier editions are available only in hardcopy.

The regional and national rates were directly age-standardized to the “world standard population” of the International Agency of Research on Cancer (11). This statistical procedure ensures that the widely different age structures across populations have been adjusted for and can be compared meaningfully.

For data on the 3 indigenous groups, we utilized specialized databases. Data for Alaska Natives are obtained from the Alaska Native Tumor Registry (ANTR), a state-wide population-based registry which has been in existence since 1969. It is currently maintained by the Alaska Native Tribal Health Consortium in Anchorage, Alaska. ANTR covers Alaska Native and American Indian patients living in Alaska at the time of diagnosis who meet eligibility criteria for health care benefits from the United States Indian Health Service and its contracted providers. Alaska Native people are comprised of 3 major groups – Eskimos (here termed Inuit), Indian and Aleut. Procedures for data collection and coding follow standards of the Surveillance, Epidemiology and End Results (SEER) Program of the National Cancer Institute (14). Further separation of Alaska Native data into Inuit (Eskimo) and Indians was performed by JK and colleagues in ANTR.

Statistics Canada operates the Canadian Cancer Registry, which receives cancer data from all provincial and territorial cancer registries and performs internal record linkage and national death clearance annually. Data on cancer cases diagnosed among permanent residents of the 3 northern Canadian territories (who generally obtain cancer care services outside the territories) are maintained by the respective health departments of Yukon, Northwest Territories (NWT) and Nunavut. Although ethnic identifiers are not included in the national registry, differentiation of Inuit, First Nations and Métis people is possible in the territorial cancer registries of NWT (15) and Nunavut (16), but is incomplete in Yukon. Although regional data for Yukon are included, First Nations-specific data from Yukon were not included in this study. Inuit-specific data are also not available for the predominantly Inuit-inhabited region of Nunavik in the province of Québec.

The Danish Cancer Registry registers cases from both Denmark and Greenland. Through data linkage with the Greenland population registry, cases occurring among individuals born in Greenland and residents in Greenland at the time of diagnosis can be identified. The use of “born in Greenland” as a proxy identifier for Greenland Inuit is a long-established practice. This is not a satisfactory approach as clearly there are Danish babies being born in Greenland and Inuit babies being born in Denmark. With increased population movements, the accuracy of the “born in Greenland” as an identifier for Greenland Inuit will be reduced over time. Previously a database of cases from 1989 to 2003 was created by JF and colleagues at the Danish Epidemiology Research Centre, Statens Serum Institut in Copenhagen (7). This database was updated for the present study.

None of the national population registries or statistical databases of the Nordic countries record ethnicity. Only a handful of studies have been published where Sami identity among study participants was specifically determined based on a variety of linguistic, genealogical and sociopolitical criteria. Regional Sami cohorts have been assembled in Norway (17) covering the period 1970–1997, Sweden (18) covering the period 1961–2003, and Finland (19) covering the period 1979–1998, which was updated to 2005 in a previous review (20). The Finnish Sami cohort was further updated to 2010 by LS and colleagues at the University of Helsinki and Finnish Cancer Registry.

Ethnic identity of patients is not recorded in health care statistics in post-Soviet Russia (21). Only one study on cancer among several Russian indigenous peoples in the Arctic has been published in English, covering the period 1977–1988 (22).

Cancer cases were classified by site in accordance with the International Classification of Diseases, 10th edition (ICD-10). Only those coded as malignant neoplasms (ICD-10 codes C00 to C96 and their equivalents in earlier editions) were included in this review, excluding benign and *in situ* neoplasms. Non-melanoma skin cancer (ICD-10 C44) was excluded, because of the inconsistency across registries in including/excluding it. By excluding it, the total number of cancer cases from all sites is more comparable, without being influenced by this common, rarely fatal and often under-reported cancer.

We did not conduct independent validation of diagnosis or classification, but accept them as reported by the official agencies. In terms of quality of incidence data, GLOBOCAN assigns grade D to Russia and grade A to all the other Arctic States (10).

## Results

### Regional variation in cancer incidence

We were able to collect cancer incidence data from all 8 Arctic States and 20 of their northernmost regions for the decade 2000–2009 (Table I). The various data sources for the countries and regions are provided in the notes to the table. Among northern regions, the highest age-standardized incidence rates are observed in Nunavut, Greenland and Alaska. Note these data refer to national and regional statistics which are not specific to indigenous people. Moreover, Nunavut and Greenland have the highest proportion of indigenous people (Inuit) in their population, accounting for more than 85%. Other regions with high proportion of indigenous people are the NWT (50%), Yukon (25%) and Alaska (20%) (2). The proportion is lower in the northern regions of Eurasia. In general, the disparities in cancer incidence between the northern regions and their national counterparts are least among the Nordic countries, but considerable between Greenland and Denmark, and between Nunavut and Canada.

**Fig. 1:**
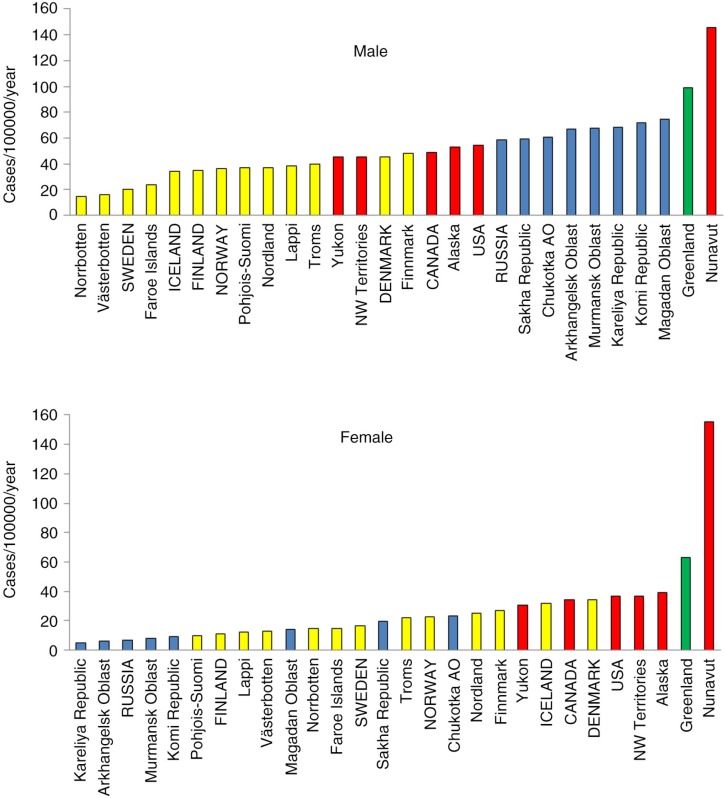
Age-standardized incidence rates of lung cancer among men and women in the Arctic States and their northern regions, 2000–2009. Note: AO=autonomous okrug. All 8 Arctic States (in capital letters) and most of their northern regions are included in the chart – **blue** refer to Russia and its northern regions, **yellow** to the Nordic countries and their northern regions, **red** to Canada and USA and their northern regions, and **green** to Greenland.

**Fig. 2:**
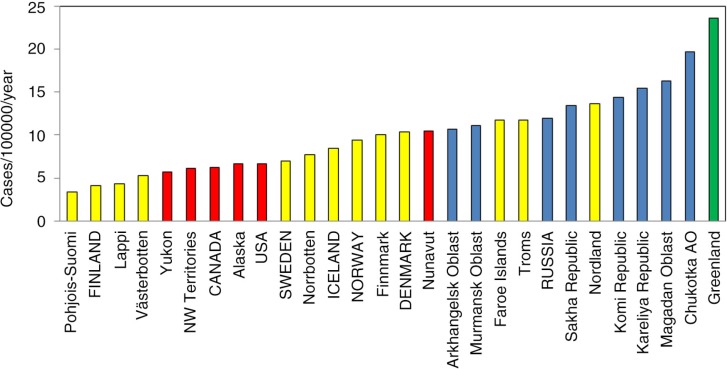
Age-standardized incidence rates of cervical cancer among women in the Arctic States and their northern regions, 2000–2009. Note: AO = autonomous okrug. All 8 Arctic States and most of their northern regions are included in the chart – **blue** refer to Russia and its northern regions, **yellow** to the Nordic countries and their northern regions, **red** to Canada and USA and their northern regions, and **green** to Greenland.

Among the different cancer sites, cancer of the lung and colon/rectum in both sexes and breast in women are the commonest in most populations. For lung cancer, Nunavut and Greenland lead all regions and countries in both men and women. Russia and its regions have high rates among men, but the opposite is true for women (Fig. 1). Greenland reports the highest incidence of cervical cancer, followed by several Russian regions (Fig. 2). Higher rates for breast cancer are found in North America and the Nordic countries, while Greenland, Nunavut and Russian regions are at the low end of the range.

### Inuit in Alaska, Canada and Greenland

Globally there are approximately 165,000 Inuit, distributed in the United States (primarily Alaska), Canada (mainly the northern territories of Nunavut and NWT) and Denmark/Greenland. There are also fewer than 2,000 Inuit in Russia, primarily in its easternmost region of Chukotka (2).

We combined the Inuit cases and populations in Alaska, NWT, Nunavut and Greenland to create a “Circumpolar Inuit” group. We obtained data from the ANTR, the territorial cancer registries of NWT and Nunavut, and the Danish Cancer Registry. Incidence data are grouped into 4 5-year periods from 1989 to 2008 (Table II). Although substantial number of Inuit also live in other American states (the “lower 48”), southern Canadian provinces and metropolitan Denmark, information on cancer occurrence in these groups is not available.

**Table II:** Age-standardized incidence rates among Circumpolar Inuit by cancer site, sex and time period

	Circumpolar Inuit (M)					Circumpolar Inuit (F)				
		
Site	89–93	94–98	99–03	04–08	1989–2008	89–93	94–98	99–03	04–08	1989–2008
Lip, oral cavity and pharynx										
Salivary glands	4.9	3.8	1.4	1.5	2.9	2.6	3.5	1.7	0.9	2.2
Mouth	3.7	2.9	2.5	0.9	2.5	2.0	3.1	0.8	2.7	2.2
Nasopharynx	14.0	14.4	9.7	15.8	13.5	5.9	7.7	7.4	9.6	7.6
Digestive organs										
Oesophagus	16.0	12.7	14.6	12.8	14.0	5.7	7.8	3.4	7.2	6.0
Stomach	30.7	24.3	27.3	28.2	27.7	12.3	7.5	8.6	15.8	11.1
Colon/rectum	42.5	49.3	54.6	78.3	56.2	39.3	51.5	69.2	67.1	56.8
Liver	8.4	7.8	8.3	9.3	8.4	3.4	4.7	3.1	5.4	4.2
Gallbladder/bile ducts	4.2	2.5	2.4	3.8	3.2	5.3	5.6	6.1	5.3	5.6
Pancreas	8.9	9.0	14.7	21.7	13.6	12.5	10.3	16.3	15.3	13.6
Respiratory and intrathoracic organs										
Nasal cavities/sinuses	0.0	0.0	0.4	1.7	0.5	0.0	0.0	0.0	0.4	0.1
Larynx	4.0	1.0	3.5	4.4	3.2	0.5	0.5	0.0	0.5	0.4
Lung	95.2	103.3	108.5	135.4	110.6	51.0	69.1	68.4	96.0	71.1
Bone and soft tissues										
Bone	2.7	1.2	0.9	1.7	1.6	0.9	1.3	0.5	0.5	0.8
Connective tissue	1.2	0.4	0.7	1.6	1.0	1.5	0.9	0.6	1.4	1.1
Skin										
Malignant melanoma skin	0.0	0.4	0.8	0.4	0.4	1.0	1.2	0.8	1.0	1.0
Breast										
Breast	0.0	0.0	0.9	1.1	0.5	42.9	60.9	55.0	78.3	59.3
Female genital organs										
Cervix uteri						26.9	19.6	17.4	20.5	21.1
Corpus uteri						3.0	1.4	4.4	7.2	4.0
Ovary						10.5	11.5	9.3	15.5	11.7
Male genital organs										
Prostate	13.1	10.5	13.4	22.7	14.9					
Testis	1.8	1.8	2.5	2.4	2.1					
Urinary tract										
Kidney	14.5	12.2	13.4	18.7	14.7	12.3	11.3	6.2	10.0	9.9
Bladder	2.6	2.5	5.1	7.5	4.4	0.9	0.8	1.9	3.2	1.7
Eye, brain and other CNS										
Eye	0.0	0.0	0.0	0.0	0.0	0.9	0.3	0.0	0.0	0.3
Brain/CNS	2.2	0.3	2.6	2.6	1.9	2.6	2.3	2.0	5.7	3.2
Endocrine glands										
Thyroid	0.0	0.4	0.5	2.6	0.9	2.1	3.0	4.0	4.3	3.4
Lymphoid/haematopoietic tissues										
Non-Hodgkin lymphoma	3.2	2.5	3.0	9.2	4.5	1.7	3.4	3.5	7.5	4.0
Hodgkin's disease	0.4	0.0	0.0	0.7	0.3	0.6	0.5	0.0	0.2	0.3
Leukaemia	1.8	5.9	2.7	3.7	3.5	3.0	2.8	1.1	6.4	3.3
Multiple myeloma	2.1	2.7	0.8	2.8	2.1	0.0	1.3	1.6	2.3	1.3
All others	25.8	31.1	36.3	44.9	34.5	37.4	39.7	34.1	41.0	38.0
All sites	304.6	302.9	331.1	436.6	343.8	288.8	333.6	327.7	431.0	345.3

**Fig. 3:**
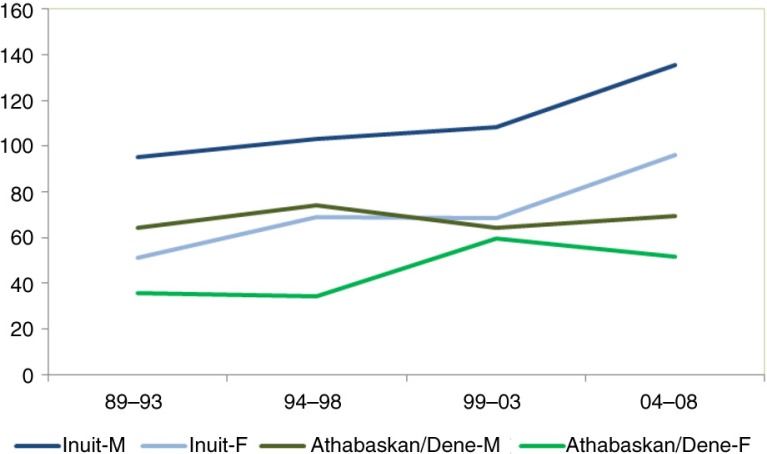
Time trend in lung cancer incidence among circumpolar Inuit and Athabaskan/Dene, 1989–2008.

**Fig. 4:**
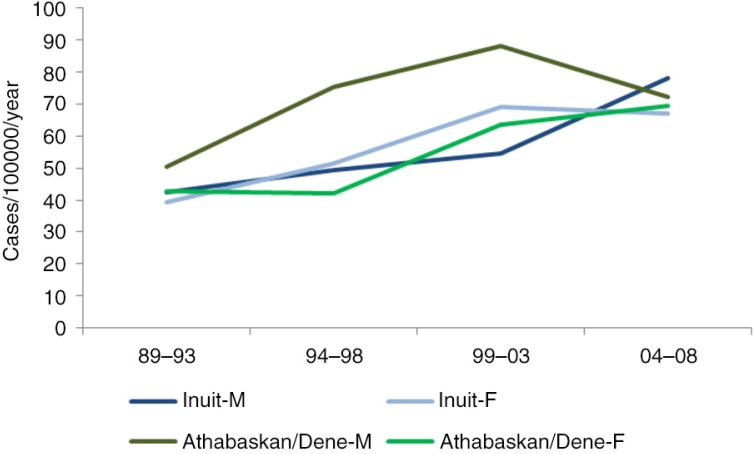
Time trend in colorectal cancer incidence among circumpolar Inuit and Athabaskan/Dene, 1989–2008.

There has been an overall increase of cancer (all sites combined) over the 4 5-year periods from 1989 to 2008. The increase is particularly marked for lung (Fig. 3), colorectal (Fig. 4) and female breast cancers (Table II). The overall risk of cancer among Inuit men and women has now “caught up” with those of non-Inuit in the United States, Canada and Denmark.

**Fig. 5:**
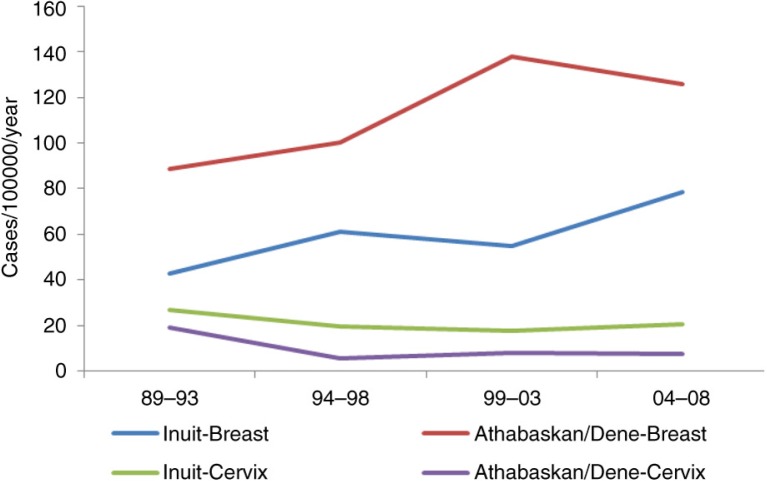
Time trend in breast and cervical cancer incidence among circumpolar Inuit and Athabaskan/Dene women, 1989–2008.

For Inuit women, breast cancer is on the rise, whereas a decline can be observed for cervical cancer (Fig. 5).

**Fig. 6:**
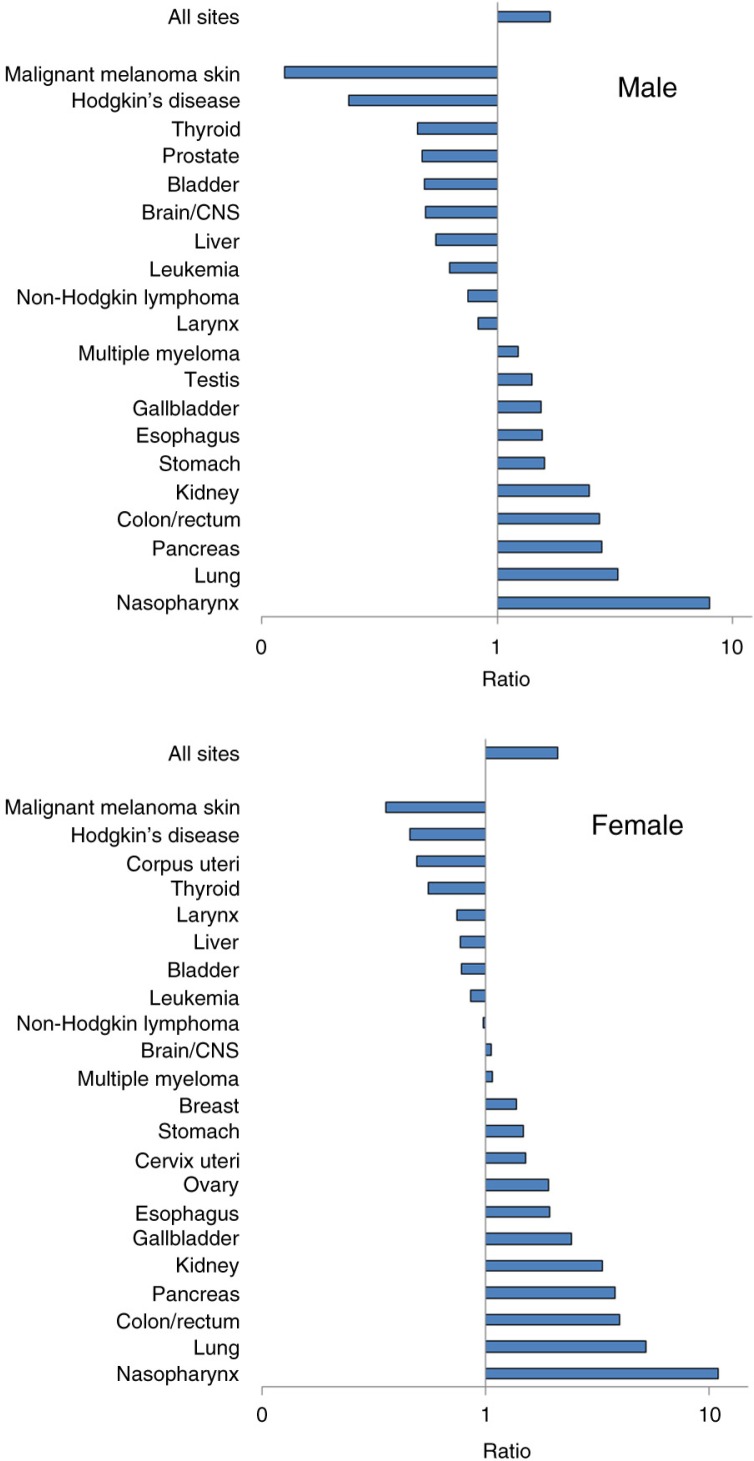
Risk of cancer by site among circumpolar Inuit relative to the GLOBOCAN world average. Note: Bars above the 1.0 line indicates excess risk among Inuit, whereas bars below the 1.0 line indicates reduced risk. Horizontal axis is in logarithmic scale.

**Fig. 7:**
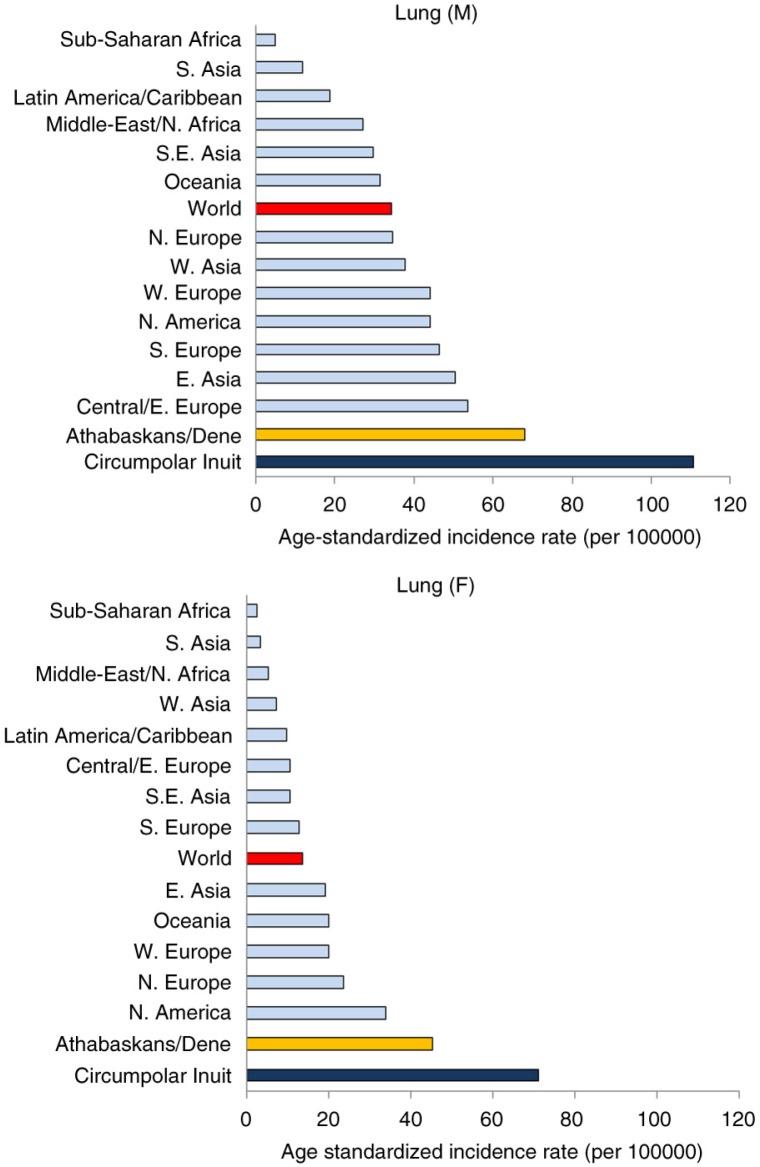
Lung cancer incidence: Circumpolar Inuit and Athabaskans/Dene compared to global regions.

To compare the risk of different cancer sites with non-Inuit, we chose the world average age-standardized rate reported by GLOBOCAN (10). Inuit are at low risk for prostate cancer, a common cancer among men. Inuit continue to be at extreme high risk for certain rare cancers such as nasopharyngeal cancer (NPC) among both men and women (Fig. 6). From a global perspective, Inuit today also have the world's highest incidence rate of lung cancer (Fig. 7).

### Athabaskans in Alaska and NWT

The Athabaskans (also spelled Athapaskans or Athabascans) are North American Indians who inhabit large swathes of the northern boreal forests of the continent. In certain regions the people self-identify as Dene. We created a group called “Athabaskan/Dene” to include members of this group who inhabit parts of Alaska and the NWT of Canada for the 4 5-year periods between 1989 and 2008 (Table III), as reported by the ANTR and the NWT Cancer Registry. While Athabaskan communities are also present in Yukon and several Canadian provinces, health data are not available by ethnicity in these jurisdictions.

**Table III:** Age-standardized incidence rates among Athabaskans/Dene by cancer site, sex and time period

	Athabaskans/Dene (M)					Athabaskans/Dene (F)				
		
Site	89–93	94–98	99–03	04–08	1989–2008	89–93	94–98	99–03	04–08	1989–2008
Lip, oral cavity and pharynx										
Salivary glands	0.0	0.0	2.3	2.2	1.1	1.5	2.0	2.6	1.3	1.8
Mouth	0.0	2.7	1.9	1.9	1.6	1.0	3.0	0.0	1.6	1.4
Nasopharynx	5.8	3.7	6.4	3.1	4.7	3.2	2.3	0.0	2.2	1.9
Digestive organs										
Oesophagus	2.5	9.5	12.4	6.3	7.6	0.0	2.1	3.5	7.5	3.3
Stomach	16.3	17.1	10.5	13.8	14.4	3.2	5.4	9.3	4.8	5.7
Colon/rectum	50.3	75.5	88.3	72.3	71.6	42.9	42.3	63.7	69.5	54.6
Liver	5.8	5.7	6.0	4.8	5.5	1.1	2.8	5.1	2.5	2.9
Gallbladder/bile ducts	2.5	3.9	8.1	6.4	5.2	4.8	1.6	7.9	1.3	3.9
Pancreas	9.0	7.3	12.0	8.9	9.3	9.4	8.5	8.9	10.4	9.3
Respiratory and intrathoracic organs										
Nasal cavities/sinuses	0.0	0.0	0.0	1.1	0.3	0.0	0.0	0.0	0.0	0.0
Larynx	5.3	4.6	2.2	2.7	3.7	0.0	0.0	2.2	0.7	0.7
Lung	64.4	74.2	64.5	69.3	68.1	35.7	34.2	59.4	51.5	45.2
Bone and soft tissues										
Bone	1.5	0.0	0.0	0.7	0.6	0.8	0.0	2.0	0.7	0.9
Connective tissue	0.0	2.2	0.7	2.7	1.4	0.0	1.1	3.5	3.5	2.0
Skin										
Malignant melanoma skin	1.9	1.1	0.0	2.1	1.3	0.7	5.2	1.3	2.0	2.3
Breast										
Breast	0.0	0.0	0.8	0.0	0.2	88.8	100.4	138.2	125.8	113.3
Female genital organs										
Cervix uteri						19.1	5.6	7.9	7.3	10.0
Corpus uteri						11.7	12.5	13.2	12.8	12.6
Ovary						12.0	9.4	7.3	11.3	10.0
Male genital organs										
Prostate	82.0	51.3	76.3	75.2	71.2					
Testis	6.1	6.0	6.3	6.3	6.1					
Urinary tract										
Kidney	11.8	17.1	21.3	13.6	15.9	9.7	8.6	12.9	14.1	11.3
Bladder	7.0	12.9	13.9	11.3	11.3	2.8	4.1	2.6	5.0	3.6
Eye, brain and other CNS										
Eye	0.0	0.7	1.2	0.9	0.7	0.0	0.0	0.0	0.0	0.0
Brain/CNS	2.8	3.3	2.5	10.2	4.7	0.0	4.1	3.9	12.2	5.0
Endocrine glands										
Thyroid	0.8	2.5	2.0	2.1	1.9	10.9	4.5	13.3	4.1	8.2
Lymphoid/haematopoietic tissues										
Non-Hodgkin lymphoma	4.5	11.5	15.1	10.3	10.3	6.4	3.5	6.8	10.1	6.7
Hodgkin's disease	0.7	0.0	1.8	1.3	1.0	0.0	1.3	0.7	0.0	0.5
Leukaemia	4.7	7.5	5.6	13.8	7.9	6.0	4.7	3.4	3.2	4.3
Multiple myeloma	5.8	1.1	4.7	5.1	4.2	4.5	1.7	10.3	7.0	5.9
All others	32.9	29.6	26.0	25.0	28.4	25.0	11.7	25.6	23.7	21.5
All sites	324.4	350.9	392.7	373.3	360.3	301.2	282.7	415.7	395.9	348.9

**Fig. 8:**
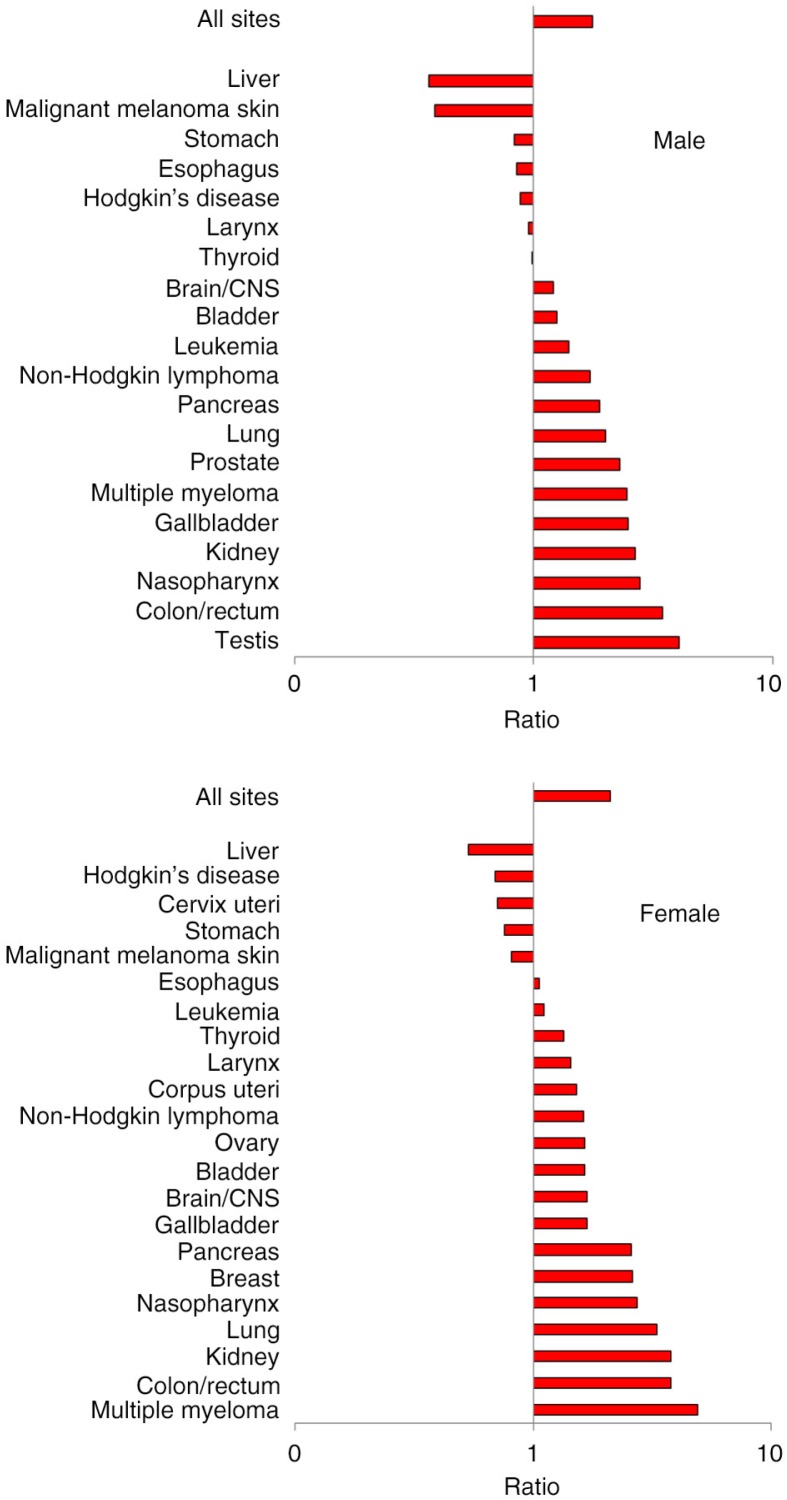
Risk of cancer by site among Athabaskans/Dene relative to the GLOBOCAN world average. Note: Bars above the 1.0 line indicates excess risk among Athabaskans/Dene, whereas bars below the 1.0 line indicates reduced risk. Horizontal axis is in logarithmic scale.

The cancer pattern among the Athabaskans sharessome similarities with the Inuit but also differs in significant respects (Fig. 8). While lung cancer incidence is still high in global terms, the Athabaskan/Dene incidence is substantially lower than that of the Inuit (Fig. 7). The incidence of colorectal cancer is higher than that of the Inuit (Fig. 4), although there is a decreasing trend in the most recent 5-year period among men. Unlike the Inuit (Fig. 6), the Athabaskan/Dene are at higher risk for prostate cancer relative to the world average (Fig. 8). Among women, the breast cancer incidence is substantially higher than that of Inuit (Fig. 5), and 3 times higher than the world average (Fig. 8). There is a downward trend in the cervical cancer rate (Fig. 5).

### Sami in the Nordic Countries

The traditional homeland of the Sami – Sápmi – covers the northern parts of Norway, Sweden, Finland and the Kola Peninsula of Russia. There is no accurate estimate of the total Sami population, which ranges from 60,000 to 110,000 (2). As the Sami data are based on 3 different research cohorts from 3 countries, we present the data separately by cohort in Table IV. Also, we can only compute relative risks comparing Sami with non-Sami in the same cohort, and not population-based rates. Hence there are no Sami rates for comparison with the Inuit, Athabaskan/Dene and GLOBOCAN regions.

**Table IV:** Risk of cancer by site among Sami in Norway, Sweden and Finland relative to non-Sami in the same regions

	Norway [1970–1997]		Sweden [1961–2003]		Finland [1979–2010]	
			
Site	M	F	M	F	M	F
Stomach	0.91	1.06	1.23*	1.53*	1.02	1.07
Colon	0.5	0.62	0.74	1.19	0.58	1.18
Rectum	1.06	0.72	0.89	1.24	0.73	0.62
Lung	0.63	0.6	0.81	0.84	0.89	0.98
Breast	–	0.85	–	1.01	–	0.38*
Ovary	–	0.88	–	1.51*	–	1.69
Prostate	0.57	–	0.76	–	0.32*	–
All sites	0.78	0.84	0.9	1.04	0.63*	0.77*

* Significantly different from unity, p<0.05.

With the exception of stomach and ovarian cancer in Sweden, the risk of cancer among both male and female Sami is not different from or significantly lower than non-Sami living in the same regions.

## Discussion

Cancer is becoming a significant public health problem in the Arctic, especially among some indigenous populations. From a global perspective, the circumpolar Inuit and Athabaskan/Dene have rates for several cancer sites that exceed all other regions in the world. An increasing trend is also evident, and represents a change from a few decades ago when the risk of cancer was generally below that of non-indigenous populations in the same region.

There are methodological limitations that pertain to the primary data sources which are beyond the control of the authors. Most but not all Arctic States have national and regional cancer registries that use internationally standardized methods of data collection and reporting. Unfortunately the availability of ethnic-specific (especially with regard to indigenous populations) is the exception rather than the rule, We were able to obtain data on 3 indigenous groups – the Inuit, the Athabaskan/Dene and the Sami, and even among these groups, not all geographical regions can be represented. Furthermore, the size of indigenous population is small in most Artic regions, although combining the same ethnic groups living in similar habitats across national borders overcomes small sample size to a certain extent.

Of the 3 indigenous groups studied, the Sami in the Nordic countries is the exception in that its cancer pattern differs only slightly from non-Sami in the same regions. Because northern Scandinavia was heavily exposed to nuclear fallout from Soviet nuclear tests in the Kola Peninsula during the 1950s and 1960s and the Chernobyl accident in 1986, concern was expressed regarding the risk of cancer among the Sami populations of Norway, Sweden and Finland, in view of the increased levels of caesium 137 in lichen, reindeer meat, and in whole-body content among reindeer herders. Studies to date showed no detectable excess of either leukaemia or thyroid cancer, which are radiation-sensitive. Indeed, the incidence of prostate, lung, breast, and colorectal cancer is lower than in the rest of the population (17–20).

The absence of significant disparity in cancer incidence between Sami and non-Sami in the Nordic countries is similar to the patterns observed for other health indicators, including mortality measures, social determinants and health behaviours (23,24).

Among the Inuit, the extreme high risk of several cancer sites – namely nasopharyngeal cancer (NPC) – continues to be observed. NPC has long been recognized as prevalent among the Inuit, which has been dubbed as a “traditional” cancer, unlike the “modern” ones such as lung and breast (25). The risk of NPC is comparable to those observed among East Asian populations. The Athabaskan/Dene are also at high risk for NPC, although to a lesser extent than the Inuit, a pattern that differs from that observed among American Indians outside Alaska (26).

While the risk of NPC among the circumpolar Inuit and Athabaskan/Dene is extremely high relative to other populations, it is still very rare. From a public health perspective the most important cancers are lung, colorectal and breast cancer.

**Fig. 9:**
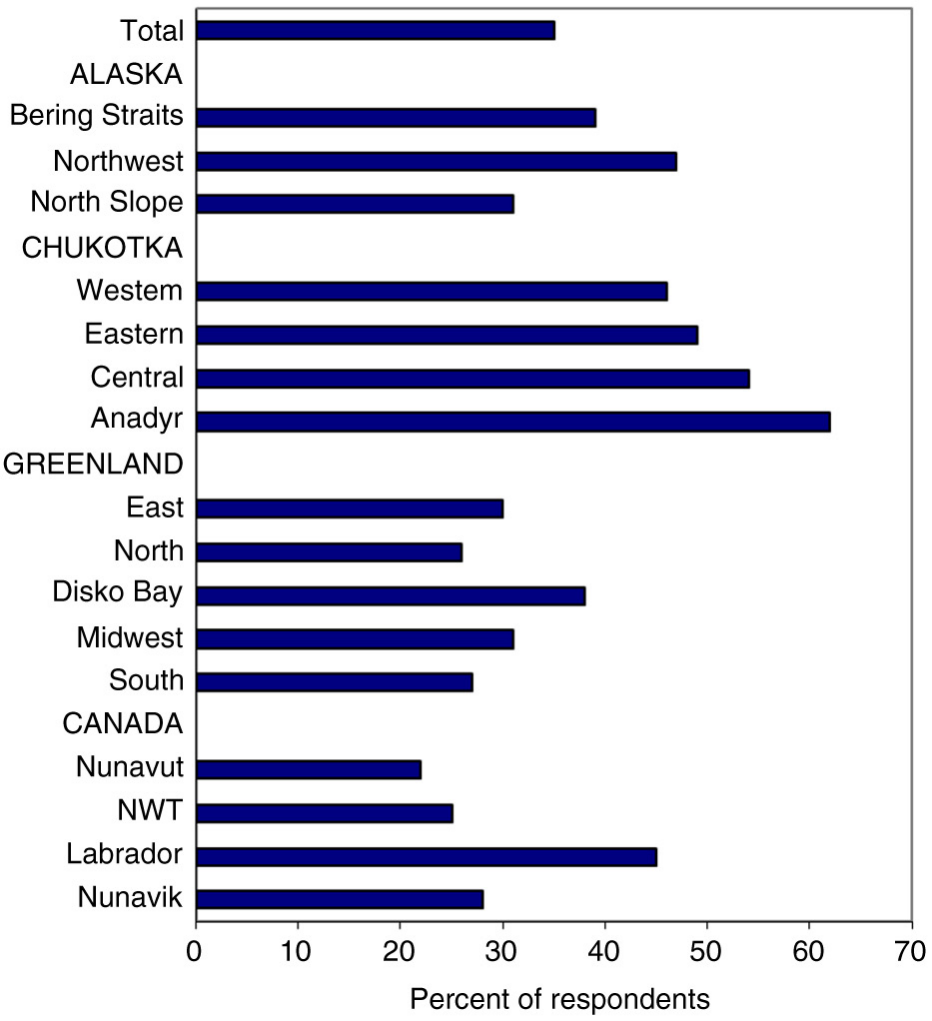
Proportion of non-smokers among the adult population in selected Arctic indigenous populations. Note: Based on data in Results Table 288 of the Survey of Living Conditions in the Arctic www.arcticlivingconditions.org [cited 2014 Dec 20].

The differential risk in lung cancer largely reflects smoking prevalence, which is extremely high among the indigenous people of Alaska, northern Canada and Greenland. For example, 39% of Alaska Natives are current smokers, compared to only 18% for the state as a whole (27). In northern Canada, almost 63% of Inuit adults are daily smokers, compared to 16% among all Canadians (28). Among the Dene in the NWT, 60% of adults are current smokers (29). Figure 9 shows the prevalence of smoking in several regional indigenous populations in the Arctic obtained by the Survey of Living Conditions in the Arctic.

The high incidence of lung cancer among men in Russia and its northern regions contrasts with the very low incidence among women. According to the 2009 Global Adult Tobacco Survey, about 55% of Russian men were daily smokers, compared to only 16% among Russian women (30).

In addition to smoking, other cancer risk factors include heavy alcohol use, low dietary intake of fruits and vegetables, obesity and physical inactivity. Changes in the population prevalence of these health determinants have occurred among Arctic populations as they experienced relatively rapid social, cultural, economic and political change (2). It should be recognized that given the long lag time for cancer to develop, even if smoking and other risk factors are dramatically reduced today, it would be decades before any impact on cancer rates would be observed.

For breast cancer, genetic susceptibility may be an important risk factor, as a *BRCA1* founder mutation has been found in the Greenlandic population, though not studied elsewhere in the Arctic (31). Another consideration is environmental contaminants such as persistent organic pollutants (POPs) including perfluorinated compounds which may increase the risk of breast cancer possibly in conjunction with certain genetic polymorphisms involved in carcinogen activation (31). POPs such as polychlorinated biphenyls and organochlorine pesticides are found at very high levels in the Arctic population (32).

The low risk of prostate cancer among Inuit contrasted with high risk among the Athabaskan/Dene has been previously observed in Alaska (33); however, the reasons are obscure. It is unlikely the result of differential screening rates. In neither group is population-wide screening with prostate-specific antigen practiced.

Cervical cancer results from infection with the human papilloma virus (HPV), which is sexually transmitted. Greenland has among the world's highest incidence rate of gonorrhoea and chlamydia infection (34), and thus the high risk of cervical cancer is not unexpected. However, Nunavut has comparable rates of sexually transmitted diseases, and yet it has a much lower burden of cervical cancer. One likely explanation is the effectiveness of Pap smear screening. Prior to 2000 Nunavut was the jurisdiction with the lowest screening participation rate in Canada. However, by 2005, the proportion of women in the 3 northern territories aged 18–69 who had at least one Pap test during the preceding 3 years exceeded the Canadian national average (35). The implementation of HPV vaccination programmes can be expected to have long term impact on cervical cancer incidence.

This study focuses only on the surveillance component of cancer control. In the face of increasing cancer risk among circumpolar populations, a variety of effective preventive strategies can be used to reduce the public health impact of cancer. This could involve primary prevention targeting health risk behaviours (smoking, diet, physical activity, etc.), certain vaccinations (against HPV and hepatitis B infection) and early detection through screening (mammography and Pap smear). Continuing epidemiological surveillance of cancer in Arctic regions will also serve the purpose of monitoring the progress and impact of interventions.
